# Exploring Race and Ethnicity Representational Inequities in Illinois Medical Schools

**DOI:** 10.1089/heq.2021.0026

**Published:** 2021-08-16

**Authors:** Nicolás O. Francone, Melissa A. Simon, Pilar Ortega

**Affiliations:** ^1^ Northwestern University Feinberg School of Medicine, Chicago, Illinois, USA.; ^2^ Department of Obstetrics and Gynecology, Northwestern University Feinberg School of Medicine and the Robert H. Lurie Comprehensive Cancer Center, Chicago, Illinois, USA.; ^3^ Departments of Medical Education and Emergency Medicine, The University of Illinois College of Medicine, Chicago, Illinois, USA.

**Keywords:** academic medicine, underrepresented in medicine, medical school admissions, racial/ethnic inequity, representational inequity, population health

## Abstract

**Purpose:** Efforts to increase U.S. medical school student diversity have lagged behind the continued growth of racial/ethnic minorities in the population. A targeted, local approach may catalyze actionable change that holds schools accountable for addressing community needs through representation. The aims of our study are to (1) analyze the student racial/ethnic profiles of allopathic and osteopathic medical schools in the diverse state of Illinois and (2) compare student race/ethnicity with that of schools' local county and primary teaching hospital patient populations.

**Methods:** Data from the Association of American Medical Colleges and American Association of College of Osteopathic Medicine were used to gather matriculated student race/ethnicity from the eight allopathic schools and one osteopathic medical school in Illinois. Representational inequity quotients (RIQs) were calculated to determine the proportion of Hispanic/Latinx, black/African American, and total underrepresented in medicine (UIM) individuals in three reference populations (U.S., county, and primary teaching hospital patient populations) relative to each medical school's student racial/ethnic profile.

**Results:** Across Illinois schools, mean RIQs were highest (showed greater inequity) when county demographics were used as the reference population as opposed to U.S. or hospital populations. For all schools individually, Hispanic county-student RIQs were higher than RIQs based on hospital population. For a majority of schools with primary teaching hospital in Cook County, hospital-student RIQs magnified representational inequity for the black population.

**Conclusions:** Using county data to evaluate medical school representation inequities may better reflect UIM representation goals than the U.S. population. Examining hospital demographics may further reveal other structural inequities relevant to medical education, such as primary teaching hospitals that are not adequately serving their surrounding communities. By evaluating RIQs on a local and hospital-population level, schools can periodically assess to what degree their student body and hospital populations represent their communities and adjust recruitment, retention, and service efforts.

## Introduction

Longstanding health care system inequities have promulgated health disparities for racial and ethnic minorities,^1^ such as Hispanic/Latinx (herein referred to as Hispanic),^2^ black/African American (herein referred to as black),^3^ American Indian/Alaskan Native (herein referred to as AIAN),^4^ and Native Hawaiian/other Pacific Islander (herein referred to as NHOPI)^5^ communities. In 2002, the National Academy of Medicine called for increasing the number of racial and ethnic minority U.S. health care professionals as a strategy to address health disparities.^6^ Later calls for diversification have cited the benefits of physician linguistic diversity,^7^ holistic admissions,^8^ and replicable examples of successful pipeline or pathway programs.^9^

According to the 2019 data, the percentage of the U.S. population who are Hispanic, black, AIAN, and NHOPI is 18.5%, 13.4%, 1.3%, and 0.2%, respectively,^10^ compared with 6.2%, 7.1%, 0.2%, and 0.1% of U.S. medical school matriculants.^11^ Physicians underrepresented in medicine (UIM) are defined by the Association of American Medical Colleges (AAMC) as those racial and ethnic populations that are underrepresented in the medical profession relative to the general population,^12^ including Hispanic, black, AIAN, and NHOPI groups. UIM physicians are more likely to practice in areas with higher density of racial minorities.^13^ Furthermore, data demonstrate better health care access, utilization, and outcomes in racially concordant patient/physician dyads^14–17^ and in language-concordant scenarios.^18^

Acknowledging the dynamic and unique health care needs of communities in a country whose racial, ethnic, and language trends continue to diversify and grow, the AAMC has proposed a “shift in focus from a national perspective to a regional or local perspective of underrepresentation.”^12^ Yet, despite known benefits and continued efforts to improve medical school diversity and implement antiracism curricula,^19^ the 2012–2017 data show there have been no significant improvements in UIM group representation relative to the evolving demography of the U.S. population.^20^ A targeted, local approach may catalyze actionable change that responds to the needs of communities in the primary service areas of a particular medical school and its teaching hospitals. Although medical school admission reform has been previously recommended, medical schools may not have been held specifically accountable for increasing the diversity of the physician workforce for their surrounding communities.

Illinois ranks 11th in state racial and ethnic diversity^21^ and 10th in percentage of population with limited English proficiency.^22^ Moreover, Illinois is home to the third largest city in the United States, Chicago, but also has a significant rural population with concerning physician shortages.^23^ Out of 19 health indicators, disparities have worsened in 14 areas for the Illinois Hispanic population and in 7 areas for the black population from 2009 to 2015.^24^ In Chicago, one of the most segregated cities, neighborhoods and hospitals vary significantly in population demographic profile and health outcomes, with a 30-year life expectancy gap across a predominantly white, affluent neighborhood and a predominantly black neighborhood only 9 miles apart.^25^

Medical schools and their teaching hospitals have an ethical responsibility to train medical students and equitably serve their local populations, but may be unfamiliar with the outcome metrics they can use to assess community needs or track their progress. The aims of our study are to (1) analyze the student racial/ethnic profiles of medical schools in Illinois and (2) compare medical school student demographics with those of their corresponding county and primary teaching hospital patient populations. Exploring local representational inequities may facilitate increased medical school accountability for ethically sound, locally responsive institutional efforts toward dismantling structural barriers for racial and ethnic minorities.

## Methods

### Data collection

We used the most recent publicly available AAMC data to view the 2020–2021 matriculated student race/ethnicity information for Illinois' eight accredited allopathic medical schools and from the American Association of Colleges of Osteopathic Medicine (2019–2020) for the one osteopathic school.

To gather county and national population data, we used the 2019 U.S. Census estimates.^26^ We used publicly available hospital data from the Illinois Health Facilities and Service Review Board to identify patient race/ethnicity data reported by the primary teaching hospitals affiliated with each medical school.^27^ We defined primary teaching hospital as the hospital where medical students at a given school complete a majority of required clinical rotations, and we identified those hospitals by reviewing clinical curricula published on each school's website. If a medical school had many clinical sites and no primary hospital could be identified, we did not calculate hospital-student representational inequity quotient (RIQ) for those schools. Since we only accessed aggregate, publicly available data, this study was determined not to constitute human subjects research and did not require Institutional Review Board approval.

### Measures and analysis

We calculated representation quotients (RQ) as applied by Lett et al., defined as the ratio of proportion of a particular subgroup among the total population of medical school matriculants relative to the corresponding proportion of that subgroup in the U.S. population.^20^ For adaptation to our locally focused study, we calculated medical school RQ with respect to three reference populations: (1) the total U.S. population, (2) local county population, and (3) hospital patient population.

After determining RQ, we derived the RIQ, our main outcome measure, by applying the inverse to the RQ value, where an RQ value of 0.25 is equivalent to an RIQ value of 4. RIQ values greater than 1 signify that a subgroup is underrepresented in medical school matriculants, which is the case for the majority of the UIM subgroups studied in this report. For example, an RIQ of 4 indicates that a particular subgroup's representation is four times greater in the reference population (e.g., the local county) than in the medical student population.

Given the low rates of AIAN and NHOPI persons in Illinois, we calculated RIQs for the Hispanic, black, and total UIM populations (the latter of which includes black, Hispanic, AIAN, and NHOPI groups). If a medical school had no matriculated students in a particular racial/ethnic subgroup, we reported the RIQ as incalculable.

## Results

### Overall results for Illinois medical schools

Five of the nine Illinois medical schools (44%) are located within Cook County, which encompasses the city of Chicago and represents the largest county population in Illinois and the second largest in the United States. Furthermore, seven of nine schools (78%) have affiliated teaching hospitals in Cook County. Table 1 displays the percentage of the population corresponding to each race/ethnicity subgroup for each Illinois medical school and the relevant reference population.

**Table 1.** Race and Ethnicity Demographics of Student, County, and Primary Teaching Hospital Patient Populations of Illinois Allopathic and Osteopathic Medical Schools Reported in 2019–2020

School	Reference population	Hispanic^a^	Black^b^	Total UIM^c^
All	United States	18.5	13.4	33.4
Illinois schools with clinical education based in the Chicago metropolitan area				
All Chicago schools	Cook County	25.6	23.8	50.2
LUC	Student	10.9	9.2	20.7
	Hospital	19.0	22.9	42.1
CCOM	Student	3.0	0.0	3.0
	Hospital	N/A^d^	N/A^d^	N/A^d^
NU	Student	11.1	7.6	20.0
	Hospital	12.3	20.3	33.4
RFU	Student	3.8	6.4	10.3
	Hospital	N/A^d^	N/A^d^	N/A^d^
RUMC	Student	10.1	7.5	17.9
	Hospital	18.3	34.8	53.5
UC	Student	10.0	18.1	28.7
	Hospital	6.6	63.2	70.2
UICOM	Student	14.1	11.1	26.7
	Hospital	23.1	52.3	75.6
Illinois schools with clinical education based outside of Chicago				
CIC	Champaign County	6.3	13.8	20.6
	Student	6.3	8.1	18.0
	Hospital	3.0	12.3	15.5
SIU	Sangamon County	2.4	13.0	15.7
	Student	5.6	13.9	19.5
	Hospital^e^	0.9	10.2	11.3
All Illinois schools	Student mean (median)^f^	8.3 (10.0)	9.1 (8.1)	18.3 (19.5)
All U.S. allopathic schools	Student mean (median)^f^	12.5 (9.6)	9.3 (7.6)	23.4 (20.1)

^a^ Hispanic refers to Hispanic/Latinx ethnicity.

^b^ Black refers to the black/African American race.

^c^ Total UIM includes individuals identifying as Hispanic, black, American Indian/Alaskan Native, and Native Hawaiian/other Pacific Islander.

^d^ Not applicable because no primary teaching hospital was identified.

^e^ SIU hospital population was calculated by averaging the patient demographics of the two primary hospitals in which students primarily complete core rotations.

^f^ Averages represent the mean (median) of the proportion of students in each UIM category per school.

CCOM, Chicago College of Osteopathic Medicine-Midwestern University; CIC, Carle Illinois College of Medicine; LUC, Loyola University Chicago Stritch School of Medicine; NU, Northwestern University Feinberg School of Medicine; RFU, Rosalind Franklin University of Medicine and Science; RUMC, Rush University Medical College; SIU, Southern Illinois University College of Medicine; UC, University of Chicago Pritzker School of Medicine; UICOM, University of Illinois College of Medicine; UIM, underrepresented in medicine.

**FIG. 1.**
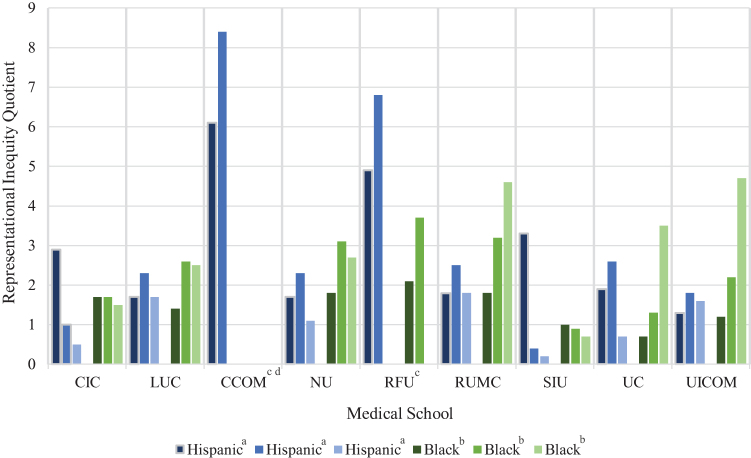
Illinois medical schools' RIQs showing representation of Hispanic and black students relative to the US, county, or primary hospital patient reference populations. ^a^Hispanic refers to Hispanic/Latinx ethnicity. ^b^Black refers to the black/African American race. ^c^Hospital RIQs not shown because the RIQ was incalculable due to the absence of a primary hospital site. ^d^RIQ for the black population not shown because value was incalculable due to the absence of black students. CIC, Carle Illinois College of Medicine; CCOM, Chicago College of Osteopathic Medicine-Midwestern University; LUC, Loyola University Chicago Stritch School of Medicine; NU, Northwestern University Feinberg School of Medicine; RFU, Rosalind Franklin University of Medicine and Science; RIQs, representational inequity quotients; RUMC, Rush University Medical College; SIU, Southern Illinois University College of Medicine; UC, University of Chicago Pritzker School of Medicine; UICOM, University of Illinois College of Medicine.

Across all Illinois schools, the mean Hispanic, black, and total UIM RIQs were highest when county demographics were used as the reference population compared with the U.S. or hospital populations (Table 2). The same was true for individual schools' county-student RIQs whose clinical education is primarily located in Cook County compared with the U.S. population, whereas the two schools located in less diverse counties yielded lower or unchanged RIQs. For all schools, Hispanic county-student RIQs were higher than RIQs based on hospital population. Table 2 shows individual schools' RIQ values using the local county and hospital patient reference populations, and Figure 1 visually compares them with that of the U.S. population.

**Table 2.** Illinois Medical Schools' Representational Inequity Quotients for Hispanic, Black, and Total Underrepresented in Medicine Subgroups Relative to County and Primary Teaching Hospital Patient Populations

School	Hispanic^a^			Black^b^			Total UIM^c^		
	US	County	Hospital	US	County	Hospital	US	County	Hospital
CIC	2.9	1.0	0.5	1.7	1.7	1.5	1.9	1.1	0.9
LUC	1.7	2.3	1.7	1.4	2.6	2.5	1.6	2.4	2.0
CCOM	6.1	8.4	N/A^d^	N/A^e^	N/A^e^	N/A^d^	11.0	16.6	N/A^d^
NU	1.7	2.3	1.1	1.8	3.1	2.7	1.7	2.5	1.7
RFU	4.9	6.8	N/A^d^	2.1	3.7	N/A^d^	3.2	4.9	N/A^d^
RUMC	1.8	2.5	1.8	1.8	3.2	4.6	1.9	2.8	3.0
SIU	3.3	0.4	0.2	1.0	0.9	0.7	1.7	0.8	0.6
UC	1.9	2.6	0.7	0.7	1.3	3.5	1.2	1.7	2.4
UICOM	1.3	1.8	1.6	1.2	2.2	4.7	1.3	1.9	2.8
All Illinois schools	2.8	3.1	1.1	>1.5^f^	>2.3^f^	2.9	2.8	3.9	1.9

^a^ Hispanic refers to Hispanic/Latinx ethnicity.

^b^ Black refers to the black/African American race.

^c^ Total UIM includes individuals identifying as Hispanic, black, American Indian/Alaskan Native, and Native Hawaiian/other Pacific Islander.

^d^ Not applicable because no primary teaching hospital was identified, so the corresponding RIQ was incalculable.

^e^ Not applicable, the absence of black student matriculants made the corresponding RIQs incalculable.

^f^ Mean RIQs for the black population were calculated excluding incalculable RIQ values, thus underestimating true mean RIQs for the black population.

RIQs, representational inequity quotients.

### Individual medical school profiles

#### Carle Illinois College of Medicine

**FIG. 2.**
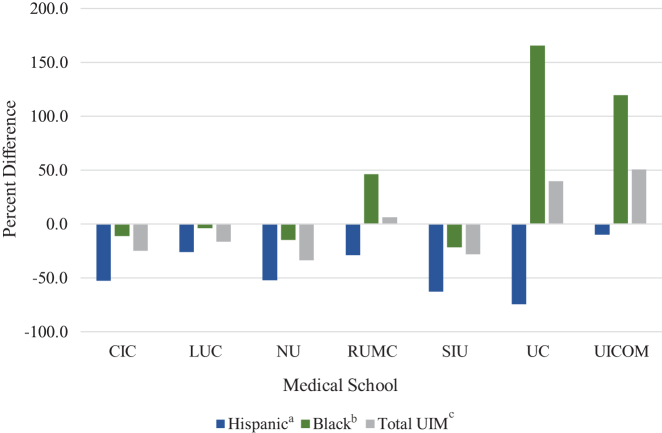
Percentage difference in Illinois medical school* representation inequity quotients when calculating representation based on hospital patient population rather than county population. ^a^Hispanic refers to Hispanic/Latinx ethnicity. ^b^Black refers to the black/African American race. ^c^Total UIM includes individuals identifying as Hispanic, black, American Indian/Alaskan Native, and Native Hawaiian/other Pacific Islander. *Two Illinois medical schools, Chicago College of Osteopathic Medicine-Midwestern University and Rosalind Franklin University of Medicine and Science are not shown because a primary teaching hospital site was not identified. UIM, underrepresented in medicine.

The Carle Illinois College of Medicine (CIC) is located in the county of Champaign, a small urban area. CIC's primary hospital has a Hispanic, black, and total UIM patient population that is less than that of the county, yielding lower RIQs (Fig. 2).

#### Loyola University Chicago Stritch School of Medicine

The Loyola University Chicago Stritch School of Medicine (LUC), located in Cook County, has a primary hospital with a Hispanic, black, and UIM patient population that is slightly less than that of the county. When LUC's primary hospital patient population is used, the RIQs change minimally across all subgroups (Fig. 2).

#### Midwestern University-Chicago College of Osteopathic Medicine

The Midwestern University-Chicago College of Osteopathic Medicine (CCOM) is located in the suburban DuPage County but offers most core clinical rotations in urban Cook County. RIQs for the black subgroup were incalculable due to the absence of black students who matriculated in the 2019–2020 school year. We were unable to calculate hospital-student RIQs due to multiple clinical sites.

#### Northwestern University Feinberg School of Medicine

The Northwestern University Feinberg School of Medicine (NU), located in Cook County, has a primary hospital with a lower Hispanic, black, and UIM patient population than that of the county. Thus, hospital-student RIQs were lower for all groups compared with county-student RIQs, with a 52% decrease in the Hispanic RIQ, nearing an RIQ of 1 (Fig. 2).

#### Rosalind Franklin University of Medicine and Science

The Rosalind Franklin University of Medicine and Science (RFU) is in Lake County, and students complete most clinical rotations in Cook County. We were unable to calculate hospital-student RIQs due to multiple clinical sites.

#### Rush University Medical College

The Rush University Medical College (RUMC), located in Cook County, has a primary hospital with a Hispanic patient population less than, a black patient population greater than, and a total UIM patient population similar to that of the county. Thus, hospital-student RIQs were lower for the Hispanic population and higher for the black population, with little change in RIQ for the total UIM (Fig. 2) compared with county calculations.

#### Southern Illinois University College of Medicine

The Southern Illinois University College of Medicine (SIU), located in Sangamon County, a small urban area, has hospitals that treat the surrounding rural communities of central and southern Illinois. Compared with using the U.S. population, calculating RIQs based on the county yielded lower RIQ values for all groups (Fig. 1). SIU has two primary teaching hospitals, both with similar patient demographics. Minority populations at the hospitals are less numerous than the population of the county. Thus, the resulting hospital-student RIQs were lower, suggesting an overrepresentation (RIQ <1) of all UIM subgroups (Fig. 2).

#### University of Chicago Pritzker School of Medicine

The University of Chicago Pritzker School of Medicine (UC), located in Cook County, has a primary hospital with a lower percentage of Hispanic and a higher percentage of black and total UIM patient populations compared with that of the county. Despite ranking first in black student representation among Illinois schools, the proportionally greater black UC hospital patient population results in a higher hospital-student RIQ (166% increase compared with county-student RIQ). Conversely, because of the hospital's lower Hispanic census, the resulting hospital-student RIQ is lower (74% decrease) compared with the county-student RIQ (Fig. 2).

#### University of Illinois College of Medicine

The University of Illinois College of Medicine (UICOM) is considered a single, urban medical school with three campuses, with the majority of students attending the Chicago campus (Cook County) and fewer students attending the Peoria (Peoria County) and Rockford (Winnebago County) campuses. AAMC demographic data from the three campuses are reported in aggregate. UICOM's primary hospital has a Hispanic population slightly less than, a black population greater than, and a total UIM population greatest of all the hospitals studied. Applying hospital patient demographics yields an RIQ of 4.7 (120% higher than using county data) for the black population. Due to similar hospital and city representation of the Hispanic population, there was little change in the RIQ for that group (Fig. 2).

## Discussion

Our study examines the extent to which Illinois medical schools' student profiles represent the racial/ethnic composition of their county and hospital patient populations. Medical schools are gateways to producing physicians for their local communities, with nearly 40% of physicians practicing in the state in which they attended medical school.^28^ Given that the UIM graduates are more likely to care for the racial/ethnic population with which they personally identify,^29,30^ knowledge of a school's degree of representational equity and purposeful recruitment of UIM students to communities with the greatest need have the potential for enhancing physician workforce diversity at the local, state, and national levels.

Our findings indicate that county characteristics most frequently revealed greater representational inequities in medical schools than U.S. population demographics, and suggest that using county data may better reflect minority representation goals. This was the case for medical schools located or affiliated with hospitals in counties with larger UIM populations, which describes the majority of Illinois schools.

Importantly, our data suggest that medical school representational inequities may sometimes be magnified or other times hidden when calculating RIQ using hospital patients as a reference. For instance, in three schools, the black patient population was higher in the hospital population than it was in the county (Table 1), resulting in magnified underrepresentation of black students as evidenced by higher hospital-student RIQs compared with county-student RIQs (Table 2). In a few cases, however, using hospital patient population hid some representational inequities. For example, for two of five schools with an identifiable primary teaching hospital in Cook County, the proportion of hospital patients who are UIM was lower than of the county population (Table 1), resulting in lower RIQs when using hospital patient populations as a reference. For the Hispanic subgroup specifically, none of the primary teaching hospitals had a Hispanic patient percentage equal or above that of the county. Differences observed in the representation of Hispanic and black populations in Illinois suggest that individual UIM subgroups may experience unique barriers to health care access and support approaches that evaluate racial/ethnic groups in a nuanced, disaggregated manner rather than viewing results for the total UIM groups only.

Our findings suggest that in addition to recruiting UIM students, teaching hospital service to the surrounding local population should be evaluated and addressed. Although medical schools may have nonprimary clinical sites where students may see higher percentages of UIM patients, the lack of community representation in primary teaching hospitals may limit student training and raises concerns about equitable access to specialty care and clinical trials. Examining hospital patient demographics is critical to ensure an ethically sound, system-wide effort to serving vulnerable local populations and reducing health disparities. However, since academic hospitals may not adequately represent the local population due to structural barriers to care for racial/ethnic minorities, hospital-student RIQs may, in some cases, give an inaccurate impression that representational equity has been achieved. Based on our study, the most accurate reference population for medical schools to use to generate representational equity goals for student admissions may be the local county population.

Our study has some limitations. Since many schools offer varied, multisite clinical learning opportunities for students, we focused our hospital population analysis to primary teaching hospital only, potentially missing other important clinical sites with different demographic profiles. In addition, some hospital patient demographic data were incomplete, with unknown values ranging (1.9–25.1%). Underreporting of race/ethnicity data is a known challenge among minority communities and may result in undercounting the percentage of Hispanic, black, and overall UIM patients in those hospitals.^31^ As a result, our reported RIQs may underestimate representational inequities.

Our findings have important actionable implications for medical schools to effectively and equitably respond to local population needs. By evaluating RIQs on a local and hospital-population level, schools can assess to what degree their student body and hospital populations represent their communities and plan appropriate changes to future recruitment, retention, and service efforts. Similarly, local needs assessments may help to direct equitable distribution of medical students who wish to use specific skills (such as language skills)^32^ or lived experiences (such as racial/ethnic identity) in areas where their skills are best matched to the needs of the population. RIQs may be an accessible, replicable methodology to help schools' accountability in reaching specific, realistic UIM representation milestones relative to local populations. Inequity quotients are a metric that can be periodically evaluated to ensure that schools make meaningful policy changes that are effective and responsive to the needs of the populations they serve.

## Abbreviations Used

AAMC: Association of American Medical Colleges
AIAN: American Indian/Alaskan Native
CCOM: Chicago College of Osteopathic Medicine
CIC: Carle Illinois College of Medicine
LEP: limited English proficiency
LUC: Loyola University Chicago Stritch School of Medicine
NHOPI: Native Hawaiian/other Pacific Islander
NU: Northwestern University Feinberg School of Medicine
RFU: Rosalind Franklin University of Medicine and Science
RIQs: representational inequity quotients
RQ: representation quotients
RUMC: Rush University Medical College
SIU: Southern Illinois University College of Medicine
UC: University of Chicago Pritzker School of Medicine
UICOM: University of Illinois College of Medicine
UIM: underrepresented in mdicine
